# Hemoptysis in Pediatric Patients

**DOI:** 10.7759/cureus.4305

**Published:** 2019-03-23

**Authors:** Ryan Naum, Brittany Speed

**Affiliations:** 1 Osteopathy, West Virginia School of Osteopathic Medicine, Lewisburg, USA; 2 Emergency Medicine, Ohio Valley Medical Center, Wheeling, USA

**Keywords:** hemoptysis, pediatrics, emergency medicine, foreign object, aspiration, respiratory infections, bronchoscopy

## Abstract

Hemoptysis is defined as the expectoration of blood or blood-tinged sputum. Blood-tinged sputum is a rare finding in the pediatric population. Finding the cause and treatment of the hemoptysis in pediatric patients is largely dependent on the history. In children, the most common causes of hemoptysis are infection and tracheostomy-related complications. Other causes include aberrant bronchial circulation, aspiration of foreign bodies, and bronchiectasis associated with cystic fibrosis. Due to the rarity of hemoptysis in pediatric patients, diagnosis and management of these patients can be difficult. It is important to refer to case reports and literature to best manage these patients.

We report a case of a 3-year-old male patient who presented to the emergency department (ED) with a one-day history of hemoptysis. He presented with his adopted mother who was unable to provide a comprehensive past medical or family history other than stating that the patient has had recurrent bronchial infections since his adoption. She stated that the patient had only one episode of hemoptysis just prior to arrival.

The patient did not appear to be in any respiratory distress and did not have any episodes of hemoptysis while in the ED. Due to his afebrile status and lack of evidence of current bleeding, the only intervention administered was an albuterol breathing treatment. He responded well to the breathing treatment and was discharged home with instructions to follow up with his primary care provider.

## Introduction

Hemoptysis is a rare complaint in the pediatric population. It is important to establish the severity of the hemoptysis, as well as the actual cause. With this information, an emergency medicine physician should be able to make accurate clinical decisions. A focused physical exam and history can often lead to the underlying diagnosis causing the hemoptysis. Chest radiographs in at least two views should be obtained in all patients presenting with hemoptysis. Fiberoptic bronchoscopy and high-resolution computed tomography (CT) may be used to further assist in the establishment of a cause of hemoptysis [1-4].

The severity of hemoptysis is dependent on the volume of blood lost. Scant hemoptysis refers to less than 5 mL of blood loss, mild-to-moderate hemoptysis refers to 6-240 mL of blood loss, and massive hemoptysis refers to more than 240 mL of blood loss [4]. Estimation of the volume of blood lost is important in the establishment of hematemesis severity. The life-threatening severity threshold is >8mL/kg every 24 hours [2].

## Case presentation

A three-year-old male presented to the emergency department (ED) with the chief complaint of one episode of hemoptysis that occurred just prior to arrival. His adopted mother stated that he had cold-like symptoms for the past few days prior to arrival, and on the day of arrival, he began to cough up blood. She brought the blood-tinged rag with her to the ED. He had vomited the night prior, as well as on the morning prior to arrival, and had diarrhea during that same time frame. His adopted mother said that the diarrhea and vomit were not blood-tinged. There had been no change to his urine output. She stated that he had a fever the night prior, as well as on the morning of presentation, with a maximum temperature of 101º F. His adopted mother said that he attends daycare, and that multiple children in his daycare had recently come down with respiratory syncytial virus (RSV).

His adopted mother stated that he was born full term with no complications. She also said that he had multiple bronchitis infections since his adoption, which she stated was at a few months of age. According to her, he is up to date on all vaccinations. Due to his adoption status, his family history was unknown.

His temperature on arrival to the ED was 98.7º F. He had a pulse rate of 131 beats per minute, a respiratory rate of 22 breaths per minute, and a blood pressure of 89/60. Physical exam revealed the presence of clear rhinorrhea as well as diffuse crackles and expiratory wheezing in all lung quadrants.

**Figure 1:**
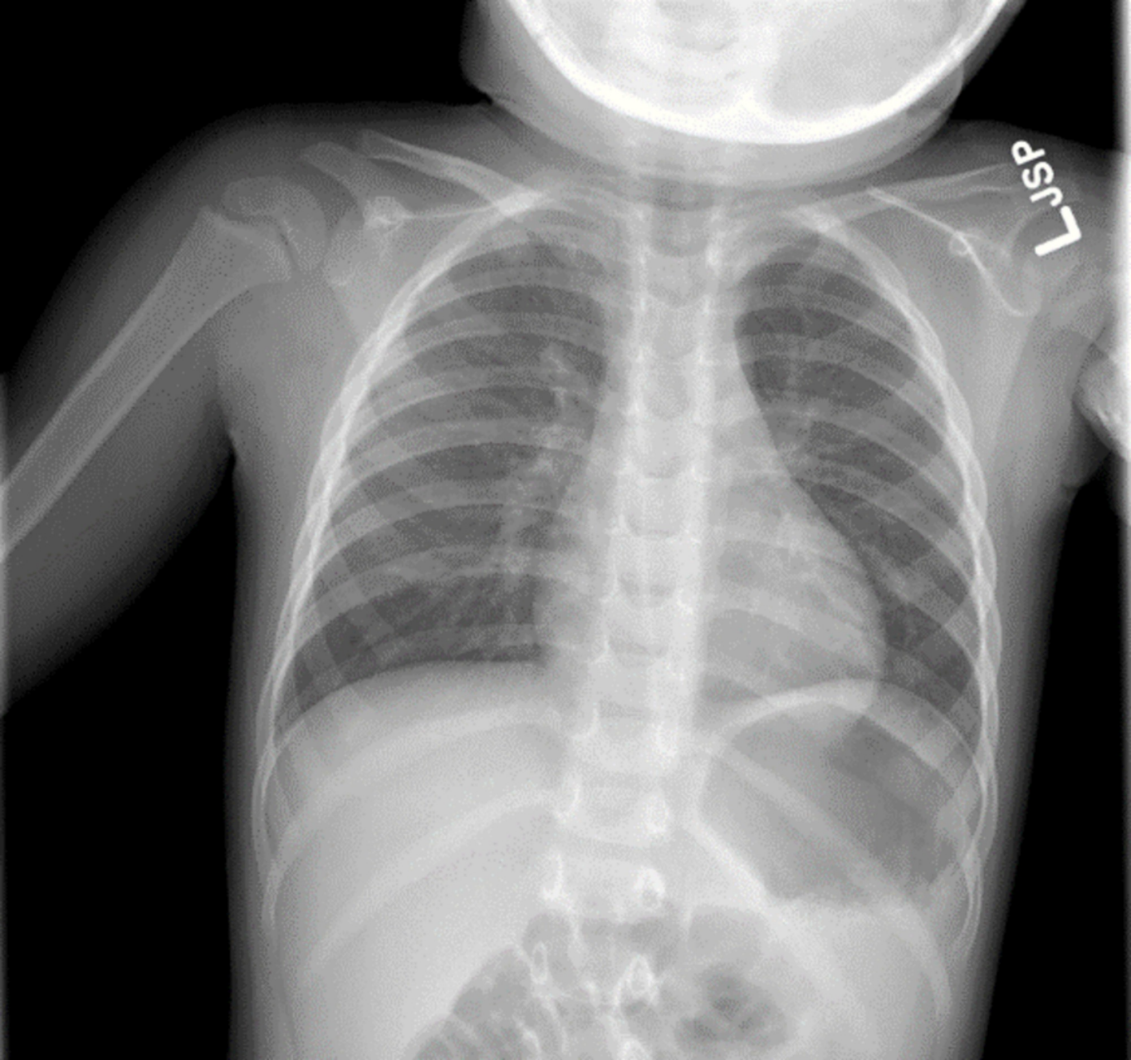
Chest radiograph in the posteroanterior (PA) view This posteroanterior chest radiograph shows no evidence of any pulmonary infiltrate or foreign body.

**Figure 2:**
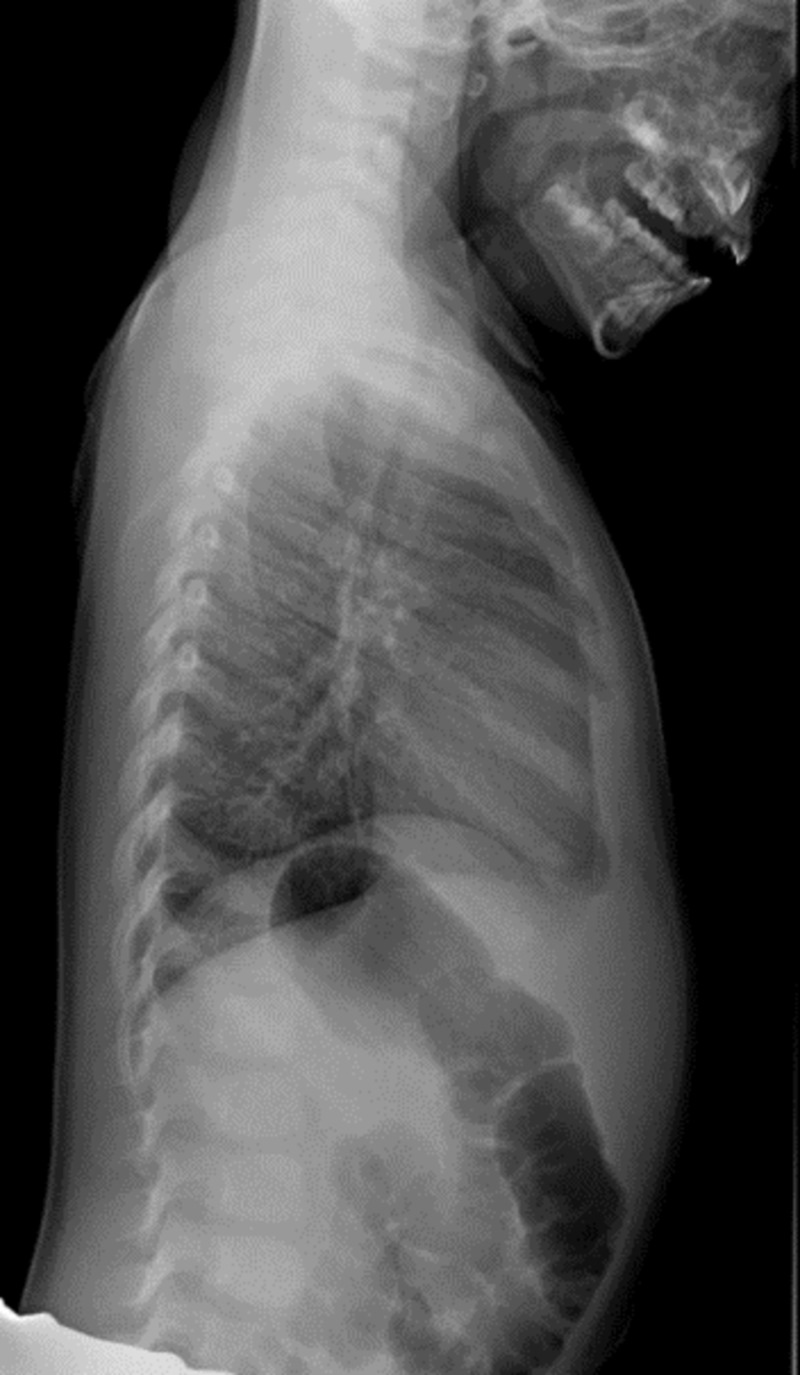
Chest radiograph in the lateral view This chest radiograph in the lateral view shows no evidence of any pulmonary infiltrate or foreign body.

A complete blood count and comprehensive metabolic panel were all within normal limits. Chest radiographs in two views were performed, and it was determined that no acute lung abnormalities or pulmonary infiltrates were present (Figures 1-2). A hemoccult test was done on the blood-tinged rag which confirmed that the substance was blood. It was estimated that the total amount of blood on the rag was significantly less than 5 mL. This placed him in the category of scant hemoptysis. 

Throughout his stay in the ED, he did not have any additional episodes of hemoptysis. An albuterol breathing treatment was performed, which he tolerated well. On re-auscultation of his lungs, the crackles and expiratory wheezing had resolved. Given his afebrile status and prior exposure to RSV, it was determined that he likely contracted RSV from his classmates. He remained stable throughout his entire stay and was discharged home with instructions to follow up with his pediatrician. It was also recommended that they follow up with a pediatric pulmonologist for evaluation of his extensive history of bronchial infections.

## Discussion

Our patient’s hemoptysis was attributed to mechanical trauma from forceful coughing, likely causing mucosal irritation and inflammation. Given his history of recurrent bronchial infections, it was suspected that he had secondary inflammatory changes that may have caused him to be at an increased risk for hemoptysis. Give that his family history was largely unknown, it was recommended that he follow up with a pediatric pulmonologist to be evaluated for possible bronchiectasis or cystic fibrosis.

Hemoptysis is broken down into three categories: scant, mild-to-moderate, and massive. In patients with scant hemoptysis, they will have lost less than 5 mL of blood. In these patients, the hemoptysis usually resolves spontaneously and is unlikely to recur. No further intervention is necessary other than observation for recurrence and appearance of other symptoms.

In patients with mild-to-moderate hemoptysis, there is a 6-240 mL blood loss. It is managed based on the cause of the hemoptysis. If foreign body aspiration is suspected, bronchoscopy should be performed for the diagnosis and removal of the aspirated object [3]. A rigid bronchoscopy is the most commonly used method for removing an aspirated foreign body. A recently published study, however, has shown that a flexible bronchoscopy using a retrieval basket is also an effective instrument for the removal of aspirated foreign bodies in pediatric patients [5].

Massive hemoptysis refers to an expectorated blood loss of more than 240 mL. These patients may need to be stabilized to prevent further bleeding. Rapid sequence intubation and mechanical ventilation with high positive end-expiratory pressure (PEEP), circulatory support, and replacement of blood products are all necessary interventions. PEEP may improve oxygenation, as well as tamponade the site of hemorrhage. If the site of hemorrhage is known, selective intubation to the unaffected lung should be done. Bilevel positive airway pressure (BiPAP) should not be used due to the risk of further aspiration of blood. Rigid bronchoscopy may be used both as an investigative tool and a treatment modality. It can be used as a manual vasoconstrictor or endobronchial tamponade, while providing ventilation of the patient. These patients may eventually require bronchial artery embolization by bronchial angiography if hemorrhaging persists [3,6].

The most common cause of hemoptysis in the pediatric population is lower respiratory infections; pneumonia and tracheobronchitis being the two most common offenders [2-6]. Hemoptysis in these patients is usually self-limiting, and all interventions should be aimed at treating the underlying infection.

Foreign body aspiration is the second most common cause of hemoptysis in pediatric patients. In the case of foreign body aspiration, the bleeding is caused by mechanical trauma to the respiratory epithelium or the ensuing inflammatory reaction, especially to vegetable matter [2]. Physicians should have a high clinical suspicion for foreign body aspiration when a pediatric patient presents with hemoptysis. Key clinical signs involve paroxysmal coughing and unexplained wheezing with normal chest radiographs. A history of choking is also highly suggestive of foreign body aspiration [2-3]. These patients should be evaluated and treated using bronchoscopy. Rigid bronchoscopy is more commonly used, but there are arguments for the use of flexible bronchoscopy as well [4-6].

Adam et al. describe a rare case of pediatric hemoptysis caused by diffuse alveolar hemorrhage [7]. In their case presentation, a 7-year-old female patient presented with scant hemoptysis which eventually became much more severe. The patient was initially well-appearing but decompensated very quickly. Chest radiographs showed the presence of large patchy infiltrates. Initial laboratory results revealed significant microcytic anemia without leukocytosis or thrombocytopenia. A bronchoscopy was performed, revealing numerous punctuate, petechial lesions on the bronchial mucosa. A lung biopsy revealed intra-alveolar hemosiderosis secondary to diffuse vascular damage. A positive perinuclear anti-neutrophil cytoplasmic antibody (p-ANCA) test along with the pulmonary findings led them to the diagnosis of diffuse alveolar hemorrhage.

Angela et al. describe an interesting case of pediatric hemoptysis caused by an atypical carcinoid tumor [8]. In their case presentation, a 6-year-old male with a history of persistent asthma presented with a persistent cough, chest pain, and a five-day history of hemoptysis. Initial labs showed increased white cell count. All other labs and cultures were normal. Physical exam showed decreased air movement on the left with left-sided tracheal deviation. Chest radiographs showed volume loss on the left with leftward mediastinal shift. A CT of his chest showed left main bronchus obstruction due to an endobronchial mass which was later established to be an atypical carcinoid tumor after biopsy.

Wegener’s granulomatosis and anti-glomerular basement membrane disease are two diagnoses that one may think of when adults present with hemoptysis but are much less common in the pediatric population. Vimal et al. describe a case of pediatric hemoptysis caused by Goodpasture’s syndrome in a 2-year-old patient [9]. They describe the difficulty in establishing the diagnosis due to its rarity in children.

## Conclusions

Hemoptysis is a symptom that rarely presents in the pediatric population. Most cases of pediatric hemoptysis are from benign causes and are self-limiting. It is important to establish a good history and do a thorough physical exam to determine the severity of the hemoptysis. Chest radiographs should be performed in all patients to evaluate for the presence of pulmonary infiltrates or foreign body. Most cases of hemoptysis in pediatric patients are caused by some sort of viral or bacterial respiratory infection, so the focus of all interventions should be managing the underlying infection.
